# Mindfulness Plus Reflection Training: Effects on Executive Function in Early Childhood

**DOI:** 10.3389/fpsyg.2018.00208

**Published:** 2018-02-26

**Authors:** Philip David Zelazo, Jessica L. Forston, Ann S. Masten, Stephanie M. Carlson

**Affiliations:** ^1^Institute of Child Development, University of Minnesota, Minneapolis, MN, United States; ^2^Learning Tree Yoga, Minneapolis, MN, United States

**Keywords:** mindfulness, reflection, executive function, intervention, preschool

## Abstract

Executive function (EF) skills are essential for academic achievement, and poverty-related stress interferes with their development. This pre-test, post-test, follow-up randomized-control trial assessed the impact of an intervention targeting reflection and stress reduction on children's EF skills. Preschool children (*N* = 218) from schools serving low-income families in two U.S. cities were randomly assigned to one of three options delivered in 30 small-group sessions over 6 weeks: Mindfulness + Reflection training; Literacy training; or Business as Usual (BAU). Sessions were conducted by local teachers trained in a literacy curriculum or Mindfulness + Reflection intervention, which involved calming activities and games that provided opportunities to practice reflection in the context of goal-directed problem solving. EF improved in all groups, but planned contrasts indicated that the Mindfulness + Reflection group significantly outperformed the BAU group at Follow-up (4 weeks post-test). No differences in EF were observed between the BAU and Literacy training groups. Results suggest that a brief, small-group, school-based intervention teaching mindfulness and reflection did not improve EF skills more than literacy training but is promising compared to BAU for improving EF in low-income preschool children several weeks following the intervention.

## Introduction

Executive function (EF) skills (cognitive flexibility, working memory, inhibitory control) are essential for goal-directed problem solving and classroom learning, and as such, they are important for kindergarten readiness (see Zelazo et al., 2017, for a review). Relations between EF and academic achievement in early childhood are robust. Results of a meta-analysis showed a mean effect size of *r* = 0.27 across 75 studies of preschool and kindergarten age children, indicating a moderate and statistically significant association (Allan et al., 2014). Children who arrive at school with well-practiced EF skills may find it easier to sit still, pay attention, remember and follow rules, control their impulses, wait their turn, and flexibly consider new ideas and different perspectives. This, in turn, may initiate a cascade of beneficial consequences: Children may learn more easily, gain confidence, enjoy going to school, and get along better with teachers and peers. Moreover, EF skills, and the reflective processes that underlie them, may jointly allow for a more fully engaged, active, and intentional form of learning (Marcovitch et al., 2008; Zimmerman, 2008). Evidence indicates that preschoolers with better EF skills do indeed learn more from a given amount of instruction and practice (Welsh et al., 2010; Benson et al., 2013; Hassinger-Das et al., 2014; Bascandziev et al., 2016).

EF skills may be especially important for children from lower socioeconomic (SES) backgrounds, in part because of the bidirectional relations between EF and stress. Children with lower SES show lower levels of EF skill, even controlling for general cognitive skills (e.g., Mezzacappa, 2004; Noble et al., 2005; Farah et al., 2006; Obradović, 2010; Masten et al., 2012). They also show higher levels of stress and stress hormones, which undermine the use of EF skills and interfere with EF development (e.g., Evans and Schamberg, 2009; Blair et al., 2011; Hostinar et al., 2014). In contrast, strong EF skills may protect against the risks associated with poverty and adversity (Obradović, 2010; Masten et al., 2012). EF skills are instrumental in regulating stress (e.g., Zelazo and Lyons, 2012; Hostinar et al., 2014; Blair and Raver, 2015), so the combination of high stress and low EF skills may pose a substantial and potentially synergistic risk to healthy neurocognitive development and adaptation more generally (Masten, 2014).

A growing body of evidence indicates that EF skills can be fostered by relatively brief interventions that provide children with opportunities to practice their developing EF skills at increasing levels of challenge (e.g., Rueda et al., 2005; Karbach and Kray, 2009; Thorell et al., 2009; Mackey et al., 2011; Tominey and McClelland, 2011; Neville et al., 2013; Weiland and Yoshikawa, 2013; Schmitt et al., 2015; see Diamond and Lee, 2011, for a review). These interventions often require children to pause momentarily and reflect before responding: in other words, to be intentional about their cognition and behavior. The repeated engagement and use of reflection and EF skills in problem solving evidently strengthens those skills, increases the efficiency of the corresponding neural circuitry, and increases the likelihood that the skills will be activated in the future (Zelazo, 2015).

According to the Iterative Reprocessing model (e.g., Cunningham and Zelazo, 2007; Zelazo, 2015), reflection involves noticing challenges, pausing, considering the options, putting things into context prior to responding, and monitoring progress toward a goal. When children respond to situations reactively, without much reflection upon what they are doing, they are more likely to show classic EF failures, such as treating a new situation as if it were an old, familiar one.

Espinet et al. (2013) provided preschool-age children with ~20 min of “reflection training” in the context of a challenging EF task, the Dimensional Change Card Sort (DCCS). Children who perseverated on this task were taught to pause before responding, reflect on the conflict inherent in the task, and formulate higher-order rules for responding flexibly: “In the color game, if it's a green pig, then it goes here; but in the shape game, that same green pig goes there.” Compared to children who received only *minimal yes/no feedback* (without practice in reflection) and to children who received *mere DCCS practice with no feedback* at all, children who received *reflection training* showed significant improvements in performance on a subsequent administration of the DCCS. Improvements were also seen on other tasks, including a measure of flexible perspective taking (a false belief task), and these behavioral changes were accompanied by predictable changes in children's brain activity, specifically a reduction in the amplitude of the N2 component in the ERP.

Moriguchi et al. (2015) also provided 3- to 5-year-old children with practice on the DCCS, but then had children teach the rules to a puppet, which demands consideration and reconsideration of what is being taught. Compared to controls, trained children showed considerable improvement in performance on the DCCS along with increased brain activity (oxygenated hemoglobin) in the left lateral parts of prefrontal cortex.

In general, EF training studies suggest it is possible to train high-level skills like reflection and cognitive flexibility, with corresponding neural changes. A consequence is that trained networks become more efficient (e.g., Hebb, 1949), so reflection and executive function occur more automatically and more quickly, providing more time for thoughtful consideration of options prior to overt action or to decision making. Although there are questions about the extent to which the benefits of EF training transfer to new situations (e.g., Diamond and Lee, 2011, for review), it has been proposed that supplementing direct EF skills training with reflection training facilitates transfer by inducing metacognitive awareness of the skills and their range of application (Zelazo, 2015).

Another, complementary approach to reflection training explicitly addresses stress reduction through mindfulness (for review see Shapiro et al., 2014). Mindfulness is a practice that entails attending to one's moment-to-moment experiences and reflecting on them in a nonjudgmental and nonreactive way. Mindfulness may be cultivated through a variety of attentional exercises, such as those included in Mindfulness Based Stress Reduction training (MBSR; Kabat-Zinn, 2003), and has been applied in a range of contexts (e.g., Segal et al., 2013; Bögels and Restifo, 2014). For example, during mindful practice, adult individuals might initially intend to focus their attention on their breathing. When they notice that their mind has wandered, they simply bring their attention back to their breathing. As with reflection and EF skills, repeated practice in becoming reflectively aware of attentional lapses presumably renders the neural networks involved in attention regulation stronger and more efficient.

A growing literature indicates that repeated engagement in mindfulness practices do indeed improve performance on measures of EF and emotion regulation (e.g., Baer, 2003; Grossman et al., 2004; Ortner et al., 2007; Tang et al., 2007; Chambers et al., 2008; Heeren et al., 2009; Zeidan et al., 2010; Schonert-Reichl et al., 2015; Zoogman et al., 2015; Lyons and DeLange, 2016; Kaunhoven and Dorjee, 2017). Improvements in emotion regulation may mediate observed reductions in social anxiety, depression, and rumination (e.g., Goldin and Gross, 2010). In addition, however, practice being nonjudgmental may promote calmness and well-being, as may focusing on the present moment (e.g., instead of ruminating over a recollected source of anxiety; Kabat-Zinn, 2003).

In children, mindfulness training often includes small group activities designed to promote sustained introspective reflection on various experiences (e.g., Flook et al., 2010). For example, to foster awareness of internal states, children might describe how different parts of their bodies feel from head to toe. Props may scaffold these exercises; for example, holding a hula hoop around their bodies and moving it up and down helps children focus attention to a zone like their shoulders, and a stuffed animal may be placed on children's abdomens to help them pay attention to their breathing as they lie down on a mat and breathe to lift the animal up and down.

In the current study, we examined the impact of a 6-week intervention for low-income preschoolers that combines reflection training and mindfulness. The combined intervention was delivered by trained teachers during 30 daily small-group sessions over 6 weeks in preschool classrooms. We expected that mindfulness activities and reflection training would provide a synergistic combination for boosting EF skills that would be well-suited to this population. Whereas mindfulness training (e.g., belly breathing; body scan) was expected to help children calm down, regulate stress, become aware of moment-to-moment experience, and sustain attention, reflection training in the context of EF games should also help children recognize when they need to “go off autopilot” and instead act deliberately, relying on their EF skills to achieve their goals. Reflection training occurred in the context of 3 EF-challenging games presented with reflection protocols designed to provide explicit consideration of their own thoughts, emotions, and behavioral tendencies in the context of goal-directed problem solving. The EF games were adapted from an EF intervention (*Ready? Set. Go!*) designed by the authors for use with homeless and highly mobile children (Casey et al., 2014). For each game, reflection protocols were designed to help teachers: scaffold children's performance on the game, adjusting the degree of challenge to maintain engagement; encourage children to notice sources of difficulty in the game and to acquire strategies for pausing, stepping back, and acting deliberately.

The active control condition (Literacy training) allowed for differentiating effects specific to the Mindfulness + Reflection training condition, controlling for receipt of an effective small-group pull-out intervention from a novel instructor for the same amount of time. We expected children in the Mindfulness + Reflection group to show greater improvement in EF at post-test and follow-up, compared to both BAU and Literacy children. Children in the Literacy condition were expected to show improvements on a standardized measure of early literacy (the Woodcock-Johnson III Letter-Word Identification subtest), compared to both BAU and Mindfulness + Reflection children. A measure of theory of mind served as a potential marker of improved awareness of self and other, and children in the Mindfulness + Reflection condition were expected to show the largest improvements.

## Methods

### Participants

The sample of 218 children (*M* = 57 months, *SD* = 3.7, range = 47–63 months) included all preschool children at two schools serving low-income families. One school in Houston, Texas, served children who were primarily Hispanic White: White = 55%; More than one = 32%, African American = 9%, Native American = 3%, Hispanic = 97.4%. The other school, in Washington, DC, served children who were African American (100%). The sample included 101 males and 117 females (53.7%; 50.5% in DC and 56.1% in Houston). The study protocol was approved by the Institutional Review Board for Human Participants at the University of Minnesota, and all parents were provided with written information about the study and received a passive (opt-out) consent form. Parents were invited to fill out a Family Information Questionnaire (FIQ) for a $10 gift card. In DC, only 28 families (29%) returned a FIQ. In Houston, 91 did so (74%). The median reported family income for both sites was $25,000–50,000 annually. See Table 1 for demographic information by location.

**Table 1:** Demographic information by location.

	**Washington, DC (29% reporting)**	**Houston (74% reporting)**
Ethnicity	100% African American	55.4% White; 32.3% more than one; 9.2% African American; 3.1% Native American
Hispanic	0%	97.4%
>3 weeks premature	21.4%	12.6%
Primary language	100% English	67.7% English; 32.3% Spanish
Bilingual	0%	68.4%
C1 gender	88.5% female	84% female
C1 age (years)	*M* = 31.93 (*SD* = 8.14)	*M* = 32.31 (*SD* = 5.91)
C1 marital status	59.3% single (never married)	13.9% single (never married)
C1 education	Mode = High school diploma	Mode = Some college
Family income last year	Median = $25,000–$50,000	Median = $25,000–$50,000

*C1, Primary caregiver*.

### Design

The sample size was determined based on the effect sizes reported in prior literature (e.g., Blair and Raver, 2014). An a priori power analysis using G^*^Power (v. 3.1; Faul et al., 2007) indicated that a sample of 200 children should provide sufficient power (>0.8) to detect a small to moderate interaction effect of time by condition assuming α = 0.05. Within each school, children were randomly assigned to Mindfulness + Reflection (*n* = 72), Literacy (*n* = 76), or Business as Usual (*n* = 68) conditions. Business as Usual involved regular classroom activities at the Houston school, and a Second Step social-emotional learning intervention (Committee for Children, 2011) at the Washington, DC school. Primary dependent measures (executive function, theory of mind, teacher-rated behavior, and academic achievement) were administered at three time points: (1) within 2 weeks prior to the start of the 6-week intervention (*Pre-test*), (2) within 2-weeks following the intervention (*Post-test*), and (3) 4–6 weeks following the Post-test (*Follow-up*). Additional measures (intelligence and school district measures) were obtained at one time point only (Pre-test or Follow-up).

### Measures

Several direct behavioral assessments were administered at pre-test, post-test, and follow-up. These included three measures of executive function, a measure of theory of mind, and a measure of early literacy. In addition, teacher ratings of children's behavior were obtained at each time point. Children's IQ was assessed at pre-test only. For one school (DC), we had access to additional data collected by the school district following the intervention.

#### Executive function

##### Head-toes-knees-shoulders (Ponitz et al., 2008)

Children were invited to play a game like “Simon Says.” Following a practice round, in part 1, they were instructed to touch their head whenever the examiner said, “touch your toes” and vice versa. If the child passed this section, then in part 2, they were given the additional instruction to touch their knees whenever the examiner said, “touch your shoulders” and vice versa (10 trials). Each trial was scored as 0 (wrong action), 1 (self-correct), or 2 (correct), with up to 20 trials, for a total possible score of 0–40. This task was designed for ages 4–7 years, has adequate test-retest reliability (0.78; Lipsey et al., 2017), and takes 5–12 min.

##### Peg tapping (Diamond and Taylor, 1996)

Children were given a wooden peg, identical to a peg held by the examiner. They were instructed to tap their peg twice when the examiner tapped his/hers once, and vice versa. Following up to two practice trials per instruction, there were 16 test trials, for a possible final score of 0–16. This task is appropriate for ages 3–5 years, has adequate test-retest reliability (0.80; Lipsey et al., 2017), and takes 5–7 min.

##### Minnesota executive function scale (MEFS; Carlson and Zelazo, 2014)

In this standardized computer tablet-based assessment designed for participants age 2 and up, children were instructed to sort virtual cards into one of two boxes on the screen according to an increasingly complex set of rules. The MEFS is nationally normed, has been used with over 30,000 children, and has adequate test-retest reliability (0.86; Carlson, 2017). Past studies have established multiple forms of criterion validity for the MEFS (e.g., Doom et al., 2014; Fuglestad et al., 2014; Hassinger-Das et al., 2014; Prager et al., 2016). Scores are automatically computed using an algorithm that combines accuracy and response time, and can range from 0 to 100. The MEFS is adaptive to children's ability and takes ~4 min to complete.

#### Theory of mind

##### Theory of mind scale (Wellman and Liu, 2004)

This measure consists of 5 brief vignettes in which children are asked to reason about the mental state of a protagonist, with increasing levels of difficulty (discrepant desire, knowledge/ignorance, discrepant belief, false belief, discrepant emotion). To receive credit for each level, they had to answer both the test and memory control questions correctly. Total scores could range from 0 to 5. The ordinal scale of this measure was confirmed in longitudinal research across the preschool period (Wellman et al., 2011).

#### Literacy

Literacy was assessed at all three time points using the *Woodcock-Johnson III (WJ-III) Letter-Word Identification subtest* (Woodcock et al., 2001). Items require children to identify and pronounce individual letters and words. Testing followed the standardized procedure with age-appropriate starting points. Raw scores were calculated based on the number of correct responses.

#### Teacher report measures

Teachers were invited to complete the Children's Behavior Questionnaire (CBQ; Very Short Form; Putnam and Rothbart, 2006), as well as the Child Behavior Rating Scale (CBRS; Bronson et al., 1990), at each time point (pre-test, post-test, and follow-up). The authors of each measure report adequate test-retest reliability. Teachers were compensated $10 for each report in the form of gift cards (up to $60 per child).

The 36-item CBQ-VSF asked parents to rate their child's temperament in a variety of situations and contexts. Twelve items each contributed to three subscales, Surgency, Negative Affect, and Effortful Control, with alphas of 0.75, 0.72, and 0.74, respectively (Putnam and Rothbart, 2006). Surgency reflects positive loadings for Impulsivity, High Intensity Pleasure, and Activity Level items, and negative loadings for Shyness items. Negative Affect reflects positive loadings for Sadness, Fear, Anger/Frustration, and Discomfort items and negative loadings for Falling Reactivity/Soothability items. Finally, Effortful Control reflects positive loadings for items indicating Inhibitory Control, Attentional Control, Low Intensity Pleasure, and Perceptual Sensitivity.

#### Additional measures

IQ was estimated using the *Stanford-Binet Early 5* (Abbreviated IQ; Roid, 2005) at one time point only (pre-test). Standard protocols and scoring methods were used.

The Washington DC group only was given Spring Assessments by the school district including: the *Peabody Picture Vocabulary Test* (PPVT-IV; Dunn and Dunn, 2007), the *Devereaux Early Childhood Assessment* (DECA; LeBuffe and Naglieri, 2012), which is a teacher-report measure of the child's Attachment/Relationships, Behavioral Concerns, Initiative, and Self-control; the *Test of Early Math Abilities* (TEMA; Ginsburg and Baroody, 2003); and the *Strategic Teaching and Evaluation of Progress* (STEP) (a direct assessment of reading readiness; Kerbow and Bryk, 2005).

### Procedure

Four local teachers (two in each city) were recruited to deliver the two active interventions, Mindfulness + Reflection and Literacy. These teachers received a full day of training at the University of Minnesota. Two teachers were trained to administer activities in the 14-lesson mindfulness curriculum (see Appendix in Supplementary Material), as well as three EF-challenging games presented with reflection protocols. Two teachers were trained to administer early literacy lessons from the Opening the World of Learning (OWL) curriculum (see www.pearsonlearning.com/microsites/owl/main.cfm; Schickedanz and Dickinson, 2005).

Children were tested by trained assessors (*n* = 3 per site) individually at their schools in spare classrooms, staff rooms, or the cafeteria. At the Houston site, the assessors were bilingual in English and Spanish and presented the tasks in the child's preferred language. Pretesting took place prior to the start of the intervention (December–January). The interventions took place in January–February. Post-testing took place in the 2 weeks immediately following the intervention, and again 4–6 weeks later.

Both active interventions, Mindfulness + Reflection and Literacy, were provided to children during 30 small-group (8–12 children) sessions (24 min each; daily for 6 weeks). Children in the Business as Usual (BAU) group remained in the classroom and engaged in regularly scheduled activities and exercises; BAU children in DC received the Second Step intervention during this period. Children in the Mindfulness + Reflection group participated in a variety of brief (e.g., 2 min) mindfulness and relaxation practices adapted for children, along with three EF-challenging games, HTKS, Bear/Dragon/Simon Says, and Mother May I? The mindfulness exercises, often involving small props (e.g., a snow globe), were introduced and repeated across sessions (see Appendix in Supplementary Material for examples). The EF games each had six levels of EF challenge that allowed instructors continually to challenge children's skills to a moderate degree. Instructors encouraged children to notice and discuss their thoughts, emotions, and behavioral tendencies. For example, in Bear/Dragon/Simon Says, children start with much easier version of Simon Says in which they are shown two puppets and first asked simply to follow the command of one puppet, then to ignore the command of another puppet, then to alternate between them, and so on through increasing levels of EF challenge (see Table 2).

**Table 2:** Adaptive levels of difficulty for bear/dragon/simon says.

Level 1: Follow Bear
Level 2: Don't Listen to Dragon (sitting on hands)
Level 3: Don't Listen to Dragon (standing)
Level 4: Bear and Dragon together with modeling
Level 5: Bear and Dragon together *without* modeling
Level 6: Reverse Bear and Dragon instructions


Intervention teachers were also given other techniques for adjusting the level of EF challenge so that the activities continued to be challenging for most if not all children in the group. For example, they were told they could use exaggerated “nice” and “mean” voices to help children remember whom to obey, remind children to “use your brain” or adopt a 3rd-person perspective, and when children become proficient at Bear/Dragon, they could try playing regular Simon Says.

The Literacy group received lessons taken from the OWL curriculum. This active control condition allowed for the identification of effects that are specific to the Mindfulness + Reflection training by providing control participants with cognitive enrichment activities, interaction with a novel teacher, and involvement in a program outside the classroom.

## Results

The initial sample included 218 children, and some data were missing from the final data set due to variations in teacher compliance (for teacher reported measures), child absences, or experimenter error. For direct behavioral measures, the final sample sizes ranged from 185 to 216 (mean *N* = 202). For teacher report measures, the final sample sizes ranged from 92 to 192 (mean *N* = 149). The majority of missing data were from teacher reports at Time 2, which came at a busy time in the Spring term. We examined how missingness on the key measures was correlated with other variables and discovered the only systematic factor was study location. Participants in DC were more likely to be missing Stanford Binet (*r* = −0.136), CBQ and CBRS at Time 1 (*r*s = −0.362), MEFS at Time 1 (*r* = −0.174), and Peg Tapping at Time 3 (*r* = −0.136), whereas participants in Houston were more likely to be missing several measures at Time 2, including Letter/Word Knowledge (*r* = 0.252), HTKS (*r* = 0.28), Theory of Mind Scale (*r* = 0.28), MEFS (*r* = 0.242), and Peg Tapping (*r* = 0.258) (all *p*s < 0.05). These patterns appeared to be due to logistical and staffing issues at the sites rather than differences in the children. Nevertheless, we included Location as a factor in the main analyses. Missing data were treated as missing using pairwise deletion in correlations and listwise deletion in repeated measures ANOVAs.

All analyses were two-tailed with alpha set to 0.05. Children in the three randomly assigned groups did not differ significantly at Pre-test on age, sex, IQ (Stanford-Binet), or any of the pre-test measures of literacy (WJ Letter/Word Knowledge), theory of mind (ToM Scale), or EF (HTKS, Peg Tapping, MEFS), all *p*s > 0.10 (see Table 3).

**Table 3:** Descriptive statistics.

	**BAU**	***N***	**Literacy**	***N***	**M+R**	***N***	**Total Sample**	***N***
Age (months)	57.51 (3.86)	68	57.28 (3.68)	76	56.96 (3.41)	74	57.24 (3.64)	218
Sex	54% F (0.50)	68	54% F (0.50)	76	54% F (0.50)	74	54% F (0.50)	218
SBIQ	95.3 (13.28)	60	98.07 (13.12)	70	100.27 (12.75)	66	97.96 (13.13)	196
HTKS T1	10.3 (12.65)	67	11.91 (13.88)	75	12.41 (14.17)	69	11.56 (13.56)	211
HTKS T2	16.98 (15.00)	62	20.04 (14.75)	68	21.28 (13.49)	64	19.47 (14.47)	194
HTKS T3	19.33 (15.33)	64	24.17 (13.87)	72	25.13 (14.50)	67	22.96 (14.69)	203
Peg Tap T1	10.71 (5.22)	68	10.83 (4.68)	76	10.63 (4.87)	68	10.73 (4.90)	212
Peg Tap T2	12.31 (4.35)	62	12.88 (3.80)	68	13.52 (3.57)	63	12.90 (3.92)	193
Peg Tap T3	13.67 (3.26)	66	13.07 (3.53)	73	14.32 (2.33)	68	13.67 (3.12)	207
MEFS T1	42.12 (12.90)	66	42.16 (11.29)	76	42.06 (11.46)	71	42.11 (11.81)	213
MEFS T2	45.52 (12.67)	61	46.97 (12.76)	64	45.20 (10.59)	60	45.92 (12.03)	185
MEFS T3	46.91 (14.82)	66	50.94 (50.94)	72	49.46 (13.39)	67	49.16 (13.84)	205
EF Comp T1	0.45 (0.20)	68	0.47 (0.19)	76	0.46 (0.19)	72	0.46 (0.19)	216
EF Comp T2	0.55 (0.21)	62	0.59 (0.19)	68	0.61 (0.19)	64	0.58 (0.19)	194
EF Comp T3	0.60 (0.20)	66	0.64 (0.18)	73	0.67 (0.17)	68	0.64 (0.19)	207
EF Rank T1	−0.04 (0.77)	68	0.02 (0.67)	76	0.00 (0.70)	72	0.00 (0.71)	216
EF Rank T2	−0.12 (0.85)	62	0.03 (0.72)	68	0.07 (0.70)	64	0.00 (0.76)	194
EF Rank T3	−0.14 (0.87)	66	0.00 (0.75)	73	0.12 (0.67)	68	0.00 (0.77)	207
ToM T1	2.78 (1.14)	68	2.70 (1.07)	76	2.58 (0.96)	72	2.69 (1.06)	216
ToM T2	3.18 (1.15)	62	3.07 (1.12)	68	2.89 (0.89)	64	3.05 (1.06)	194
ToM T3	3.2 (1.16)	65	3.33 (0.97)	73	3.06 (1.14)	67	3.20 (1.09)	205
Literacy T1	10.05 (4.23)	66	9.33 (4.35)	75	9.74 (4.93)	70	9.69 (4.50)	211
Literacy T2	13.32 (5.70)	59	12.63 (3.92)	68	13.32 (4.80)	60	13.07 (4.81)	187
Literacy T3	15.94 (6.53)	65	14.99 (5.18)	68	16.23 (6.24)	65	15.71 (5.99)	198
CBQ EC T1	4.99 (0.68)	60	5.08 (0.82)	66	4.97 (0.88)	66	5.02 (0.80)	192
CBQ EC T2	4.91 (0.79)	31	5.34 (0.91)	30	5.02 (0.82)	31	5.09 (0.85)	92
CBQ EC T3	4.98 (0.94)	49	5.12 (0.96)	50	5.03 (0.73)	49	5.04 (0.88)	148
CBQ Srg T1	4.48 (1.25)	60	4.34 (1.14)	66	4.09 (1.23)	66	4.3 (1.21)	192
CBQ Srg T2	4.71 (1.32)	31	4.22 (1.16)	30	4.33 (1.33)	31	4.42 (1.28)	92
CBQ Srg T3	4.71 (1.28)	49	4.34 (1.10)	50	4.36 (1.19)	49	4.47 (1.20)	148
CBQ NA T1	3.24 (1.19)	60	3.62 (1.16)	66	3.45 (1.23)	66	3.45 (1.20)	192
CBQ NA T2	3.21 (1.17)	31	3.57 (1.49)	30	3.17 (0.88)	31	3.32 (1.20)	92
CBQ NA T3	3.53 (1.12)	49	3.78 (1.19)	50	3.64 (1.12)	49	3.65 (1.14)	148
CBRS T1	37.38 (9.07)	60	37.18 (8.22)	66	36.61 (8.47)	66	37.05 (8.54)	192
CBRS T2	36.76 (8.38)	50	37.24 (8.45)	51	36.72 (7.89)	50	36.91 (8.19)	151
CBRS T3	36.73 (9.13)	49	38.14 (8.97)	50	36.82 (7.82)	50	37.23 (8.62)	149

*BAU, Business as Usual; M + R, Mindfulness + Reflection; SBIQ, Stanford-Binet IQ; HTKS, Head Toes Knees Shoulders; Peg Tap, Peg Tapping; MEFS, Minnesota Executive Function Scale; ToM, Theory of Mind Scale; Literacy, Woodcock-Johnson III Letter-Word Identification subtest; CBQ, Children Behavior Questionnaire; EC, Effortful Control; Srg, Surgency; NA, Negative Affect; CBRS, Child Behavior Rating Scale*.

Correlations among all study variables at Pre-test are shown in Table 4. IQ was moderately correlated with several measures of EF, ToM, and Literacy, as expected. The three EF measures (HTKS, Peg Tapping, and MEFS) were moderately correlated with one another (showed intra-individual reliability over time), thus we computed composite EF scores for each time point, by averaging the proportion scores on each EF task (proportion out of 40 on HTKS, out of 16 on Peg Tapping, and out of 100 on MEFS), yielding an EF score (0–1.0) for Pre-test, Post-test, and Follow-up for each individual. This method maximized our *N* for the overall EF analyses by accommodating missing data on a single EF measure. Data on one or more EF tasks were missing for 7% of participants.

**Table 4:** Bivariate correlations among pre-test (time 1) measures.

	**Age (months)**	**SBIQ**	**HTKS T1**	**Peg Tap T1**	**MEFS T1**	**ToM T1**	**Literacy T1**	**CBQ EC T1**	**CBQ Srg T1**	**CBQ NA1**
Age (months)	1									
SBIQ	−0.15^*^	1								
HTKS T1	0.16^*^	0.21^**^	1							
Peg Tap T1	0.16^*^	0.12^∧^	0.40^***^	1						
MEFS T1	0.007	0.27^***^	0.24^***^	0.11	1					
ToM T1	−0.02	0.25^***^	0.38^***^	0.23^**^	0.23^**^	1				
Literacy T1	0.09	0.14^∧^	0.29^***^	0.28^***^	0.10	0.14^∧^	1			
CBQ EC T1	0.02	0.22^**^	0.22^**^	0.13^∧^	0.23^**^	0.32^***^	0.36^***^	1		
CBQ Srg T1	−0.02	−0.59	0.09	0.07	0.02	0.03	0.09	−0.08	1	
CBQ NA T1	0.08	−0.02	−0.10	−0.12^∧^	−0.04	−0.004	−0.10	−0.27^***^	0.001	1
CBRS T1	0.28^***^	0.18^*^	0.31^***^	0.32^***^	0.27^***^	0.12	0.27^***^	−0.67^***^	−0.06	−0.20^**^

p < 0.10;

* p < 0.05;

** p < 0.01;

*** *p < 0.0001; SBIQ, Stanford-Binet IQ; HTKS, Head Toes Knees Shoulders; Peg Tap, Peg Tapping; MEFS, Minnesota Executive Function Scale; ToM, Theory of Mind Scale; Literacy, Woodcock-Johnson III Letter-Word Identification subtest; CBQ, Children Behavior Questionnaire; EC, Effortful Control; Srg, Surgency; NA, Negative Affect; CBRS, Child Behavior Rating Scale*.

**Figure 1:**
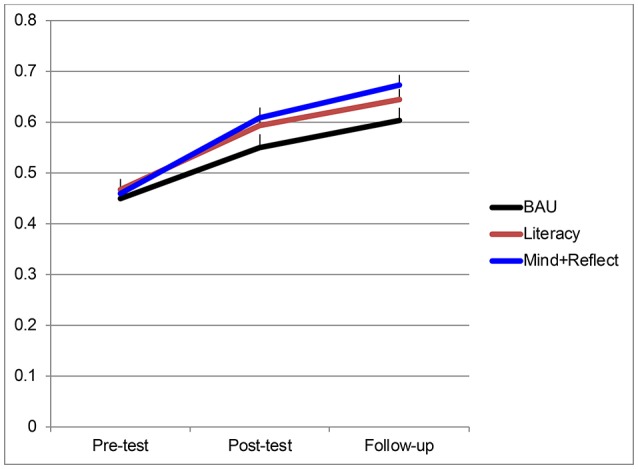
Performance on the EF composite as a function of time and condition. Bars represent standard errors. BAU, Business as Usual; Mind + Reflect, Mindfulness plus Reflection.

Next, we examined effects of the interventions on EF composite scores. As shown in Table 5, there was a highly significant linear effect of time, indicating that most children improved over the course of the study, from Pre-test to Post-test to Follow-up. There was no effect of Condition, and no interaction effect (Figure 1). In planned contrasts, however, the Mindfulness + Reflection group outperformed the BAU group (*p* < 0.05) whereas the Literacy group did not do significantly better than BAU (*p* = 0.173). Follow-up tests showed this advantage for the Mindfulness + Reflection group was a trend at the immediate post-test but significant at the delayed post-test, 4–6 weeks after the intervention was completed.

**Table 5:** Results of the repeated measures mixed ANOVAs.

**Dependent variable**	**Time**	**Condition**	**Location**	**Time × condition**	**M + R vs. BAU**
Composite EF	*F*_(1, 177)_ = 143.61, *p* < 0.0001, η*_*p*_*^2^ = .45	*F*_(2, 177)_ = 2.24, *p* = 0.11, η*_*p*_*^2^ = 0.03	*F*_(1, 177)_ = 1.06, *p* = 0.31, η*_*p*_*^2^ = 0.01	*F*_(2, 177)_ = 1.06, *p* = 0.35, η*_*p*_*^2^ = 0.01	*p* = 0.039 T2: *t*_(1, 124)_ = −1.66, *p* = 0.10 T3: *t*_(1, 132)_ = −2.16, *p* = 0.03
HTKS	*F*_(1, 172)_ = 75.82, *p* < 0.0001, η*_*p*_*^2^ = 0.31	*F*_(2, 172)_ = 2.68, *p* = 0.07, η*_*p*_*^2^ = 0.03	*F*_(1, 172)_ = 1.64, *p* = 0.20, η*_*p*_*^2^ = 0.01	*F*_(2, 172)_ = 1.05, *p* = 0.35, η*_*p*_*^2^ = 0.01	*p* = 0.04 T2: *t*_(1, 124)_ = −1.69, *p* = 0.09 T3: *t*_(1, 129)_ = −2.23, *p* = 0.03
Peg tap	*F*_(1, 174)_ = 67.53, *p* < 0.0001, η*_*p*_*^2^ = 0.28	*F*_(2, 174)_ = 1.11, *p* = 0.33, η*_*p*_*^2^ = 0.01	*F*_(1, 174)_ = .08, *p* = 0.78, η*_*p*_*^2^ = 0.00	*F*_(2, 174)_ = 0.34, *p* = 0.71, η*_*p*_*^2^ = 0.00	*p* = 0.18
MEFS	*F*_(1, 167)_ = 39.04, *p* < 0.0001, η*_*p*_*^2^ = 0.19	*F*_(2, 167)_ = 0.22, *p* = 0.80, η*_*p*_*^2^ = 0.00	*F*_(1, 167)_ = 12, *p* = 0.73, η*_*p*_*^2^ = 0.00	*F*_(2, 167)_ = 1.50, *p* = 0.23, η*_*p*_*^2^ = 0.02	*p* = 0.77
ToM scale	*F*_(1, 178)_ = 27.93, *p* < 0.0001, η*_*p*_*^2^ = 0.14	*F*_(2, 178)_ = 0.61, *p* = 0.544, η*_*p*_*^2^ = 0.01	*F*_(1, 178)_ = 7.96, *p* = 0.005, η*_*p*_*^2^ = 0.04	*F*_(2, 178)_ = 0.71, *p* = 0.493, η*_*p*_*^2^ = 0.01	*p* = 0.34
Literacy	*F*_(1, 161)_ = 231.36, *p* < 0.0001, η*_*p*_*^2^ = 0.59	*F*_(2, 161)_ = 0.37, *p* = 0.69, η*_*p*_*^2^ = 0.005	*F*_(1, 161)_ = 6.25, *p* = 0.01, η*_*p*_*^2^ = 0.04	*F*_(2, 161)_ = 0.71, *p* = 0.493, η*_*p*_*^2^ = 0.01	*p* = 0.91
CBQ effortful control	*F*_(1, 84)_ = 0.52, *p* = 0.47, η*_*p*_*^2^ = 0.006	*F*_(2, 84)_ = 2.56, *p* = 0.08, η*_*p*_*^2^ = 0.06	*F*_(1, 84)_ = 1.16, *p* = 0.29, η*_*p*_*^2^ = 0.014	*F*_(2, 84)_ = 0.87, *p* = 0.42, η*_*p*_*^2^ = 0.02	*p* = 0.53
CBQ surgency	*F*_(1, 84)_ = 5.08, *p* = 0.027, η*_*p*_*^2^ = 0.057	*F*_(2, 84)_ = 1.17, *p* = 0.316, η*_*p*_*^2^ = 0.027	*F*_(1, 84)_ = 0.17, *p* = 0.68, η*_*p*_*^2^ = 0.002	*F*_(2, 84)_ = 1.54, *p* = 0.22, η*_*p*_*^2^ = 0.035	*p* = 0.27
CBQ negative affect	*F*_(1, 84)_ = 0.6, *p* = 0.44, η*_*p*_*^2^ = 0.007	*F*_(2, 84)_ = 0.5, *p* = 0.611, η*_*p*_*^2^ = 0.012	*F*_(1, 84)_ = 6.52, *p* = 0.012, η*_*p*_*^2^ = 0.072	*F*_(2, 84)_ = 2.1, *p* = 0.129, η*_*p*_*^2^ = 0.048	*p* = 0.796
CBRS	*F*_(1, 143)_ = 2.94, *p* = 0.089, η*_*p*_*^2^ = 0.02	*F*_(2, 143)_ = 0.20, = 0.822, η*_*p*_*^2^ = 0.003	*F*_(1, 143)_ = 0.65, *p* = 0.421, η*_*p*_*^2^ = 0.005	*F*_(2, 143)_ = 0.38, *p* = 0.687, η*_*p*_*^2^ = 0.005	*p* = 0.874

*M + R, Mindfulness + Reflection; BAU, Business as Usual; SBIQ, Stanford-Binet IQ; HTKS, Head Toes Knees Shoulders; Peg Tap, Peg Tapping; MEFS, Minnesota Executive Function Scale; ToM, Theory of Mind; Literacy, Woodcock-Johnson III Letter-Word Identification subtest; CBQ, Children's Behavior Questionnaire; CBRS, Child Behavior Rating Scale*.

**Figure 2:**
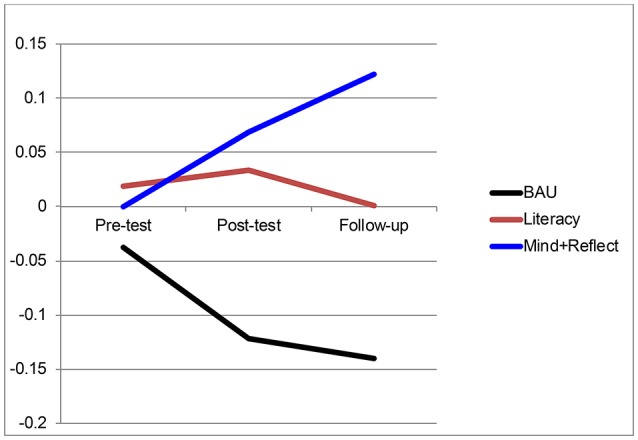
Standard scores (*z*) on the EF composite as a function of time and condition. BAU, Business as Usual; Mind + Reflect, Mindfulness plus Reflection.

Given the substantial growth in EF shown by the whole preschool sample, we examined the *rank order* of participants at each time point as a function of group assignment, using *z*-scores for the EF Composite (which resets the mean to 0 at each time point). As illustrated in Figure 2, children's ranks improved considerably for the Mindfulness+ Reflection group, whereas they declined for the BAU group and remained stable for the Literacy group. At Follow-up, the difference between Mindfulness + Reflection and BAU was significant, *p* < 0.05.

### Individual EF task analysis

**Figure 3:**
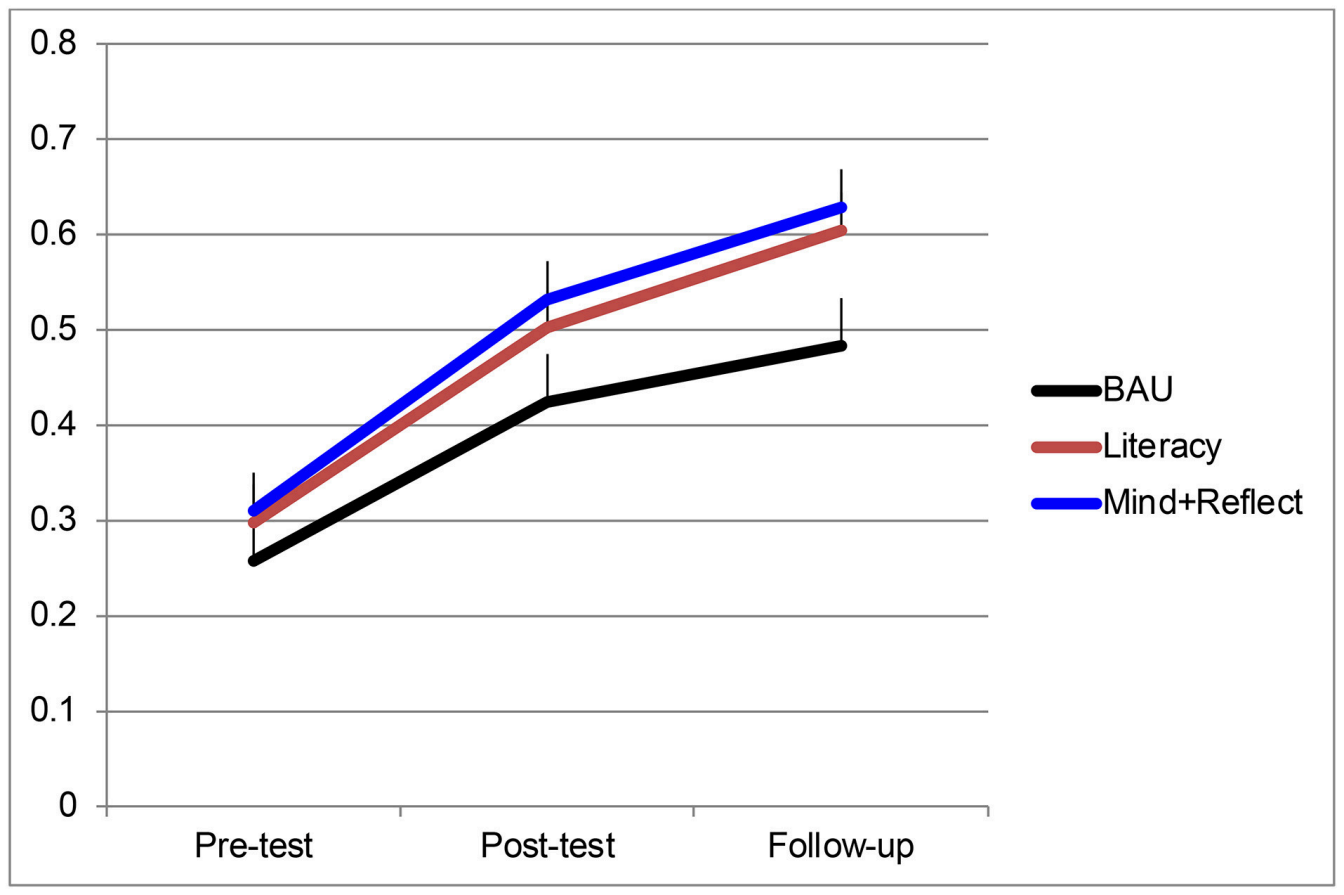
HTKS performance as a function of time and condition. Bars represent standard errors. BAU, Business as Usual; Mind + Reflect, Mindfulness plus Reflection.

In the HTKS task, there was a significant linear effect of time and a marginally significant effect of condition. Although there was no interaction between time and condition, planned contrasts revealed that the Mindfulness + Reflection group performed significantly better than the BAU control group (Figure 3). *Post-hoc t*-tests showed the difference in performance was significant at Follow-up, *t*_(129)_ = −2.23, *p* = 0.028. The Literacy training group also trended toward superior performance compared to BAU overall, *p* = 0.062, but was not significantly different from BAU at any given time point. There was a Time × Location interaction, in which the Houston sample improved more on the HTKS over time than did the DC sample, *F*_(1, 172)_ = 18.4, *p* < 0.0001, η_*p*_^2^ = 0.10.

On Peg Tapping, there was a significant linear and quadratic effect of time, but no effect of condition and no interaction (Table 5). Although the overall difference between Mindfulness + Reflection and BAU was non-significant, there was a trend at Post-test 1, *t*_(123)_ = −1.71, *p* = 0.09. There also was a significant quadratic interaction effect of Time × Location, *F*_(1, 174)_ = 6.46, *p* = 0.012, η_*p*_^2^ = 0.04, such that the Houston sample improved more from Pre-test to Post-test 1 than did the DC sample.

On the MEFS, there was again a significant linear effect of time, no effect of condition or location, and no interactions (Table 5). In contrast to HTKS and Peg Tapping, there was no evidence of an advantage for the Mindfulness + Reflection group at any time point.

### Other measures

For the Theory of Mind Scale, there was no effect of condition or any interactions involving condition. There was a significant effect of location, however, in which children in the Washington, DC sample performed significantly better overall than children in the Houston sample.

Analysis of the WJ Letter-Word Identification test showed a highly significant linear effect of time, but no effect of condition or Time × Condition interaction. The DC sample had higher literacy scores than the Houston sample overall, as might be expected given the high rate of English Language Learner status in the latter group.

For the Washington DC school only, children were administered standardized assessments by the school district, following completion of the intervention period. A MANOVA with planned contrasts found no significant effects of Condition. Planned contrasts showed a trend for the Mindfulness + Reflection group, *M*_(31)_ = 0.68, *SD* = 0.87, doing better than the BAU group, *M*_(31)_ = 0.29, *SD* = 0.90, on the STEP (a reading readiness assessment), *p* = 0.087. (Note that scores on this measure ranged from −1 to +2.)

Teachers reported on children's behavior observed in the classroom at all three time points, although several children did not have complete data. Results for the repeated measures ANOVAs are shown in Table 5. On the CBQ Effortful Control subscale, there was a main effect of condition, with the Literacy group being rated higher than the other two groups at all time points. There was no difference between Mindfulness + Reflection and BAU on teacher ratings of Effortful Control. On the CBQ Surgency subscale, ratings generally increased over time, but this did not interact with condition, and there was no difference between M+R and BAU. On the CBQ Negative Affect subscale, there was a significant effect of location, with the children in Houston being rated higher in Negative Affect than those in Washington, DC. This did not differ by condition, but it did interact with time, *F*_(1, 84)_ = 4.56, *p* = 0.038, η_*p*_^2^ = 0.05, such that ratings in the two locations became more similar over time. There was no difference between the Mindfulness + Reflection and BAU conditions. Lastly, on the Children's Behavior Rating Scale, there was a marginal effect of time (scores increasing) but this did not interact with condition and there was no difference between the Mindfulness + Reflection and BAU groups.

## Discussion

The aim of this study was to test the effectiveness of an intervention designed to improve EF skills in preschool children at-risk for school failure. The 6-week small group pull-out intervention was comprised of mindfulness (to reduce stress and increase sustained attention) and reflection (to increase meta-cognition and verbal self-regulation in the context of goal-directed problem solving). A well-established pre-literacy curriculum served as an active control condition. At Pre-test, there were no differences among conditions on any of the relevant variables (all *p*s > 0.10).

Teacher ratings of behavior showed few condition differences and no Condition × Time interactions indicating intervention effects. Direct behavioral assessments of EF, however, revealed some intervention effects. All groups showed improvement in EF skills (measured behaviorally) over the 5-month span of the study, which was expected because the preschool period is marked by particularly rapid EF development (Carlson et al., 2013). The Mindfulness + Reflection group did not show larger improvements in EF than children in the Literacy group. However, planned contrasts showed that the Mindfulness + Reflection group (only) significantly outperformed the BAU group, with the differences most pronounced at Follow-up. This effect was most clearly seen when examining the *rank order* of participants at each time point as a function of group assignment. Children's ranks went up markedly over time for the Mindfulness + Reflection group, whereas they declined for the BAU group and remained stable for the Literacy group. Thus, while all children showed improved EF skills, children in the Mindfulness + Reflection group climbed to the top of the class and those receiving BAU occupied the lowest ranks by the end of the study. In contrast, the Literacy group (active control) did not differ from BAU on EF at any time point. In future research, it will be important to investigate the longer-term stability of intervention effects on EF, as well as how improvements in EF may predict improvements in children's academic achievement.

It is notable that of the three EF outcome measures, HTKS showed the strongest results favoring the Mindfulness + Reflection intervention. This task also bears the strongest resemblance to the reflection activities that were repeated throughout the curriculum (modified HTKS and Bear/Dragon), suggesting a near-transfer effect. Peg-tapping, which also requires children to explicitly do an opposite motor activity, showed positive results for Mindfulness + Reflection in the immediate post-test only. The MEFS could be considered a farther transfer task because it was not directly trained. Similarly, theory of mind, which requires shifting mental perspectives, was not improved by either intervention. Thus, we found a transfer gradient effect in which the activities most similar to the training showed the greatest benefit, consistent with other EF interventions to date (Diamond and Lee, 2011).

Children at the Houston site showed larger improvements on two measures of EF (HTKS and Peg Tapping) than children at the DC site, and the English-speaking DC sample had higher literacy scores than the bilingual Houston sample overall. Location differences are difficult to interpret because the two sites differed in a variety of ways, but these findings highlight the need to consider the range of contexts in which particular interventions are most effective. One possible explanation for the site differences is that parents of children in the Houston site may have been more engaged. Whereas only 29% of the DC families returned a Family Information Questionnaire (FIQ), 74% of the Houston families did so.

Overall, results suggest that a brief small-group school-based intervention that teaches mindfulness and reflection in the context of goal-directed problem solving is promising for improving EF skills in pre-school age, low-income children, and that the effects of this intervention on EF may become more pronounced during in the weeks following the intervention. The finding that effects become more pronounced following the intervention, a “sleeper effect,” is consistent with the idea that these skills require time for consolidation, independent practice, or generalization to the context of the EF assessments (Hermida et al., 2015).

The importance of EF in early childhood education is increasingly widely recognized, and the participating schools already place a lot of emphasis on self-control. For this reason, it is possible that the baseline rate of EF development in this sample was already very high. The MEFS measure is standardized and, in fact, the children in our study performed at the 47th percentile nationally, whereas low-income children score at the 38th percentile on average (Carlson, 2017). It is possible, therefore, that this RCT subjected the Mindfulness + Reflection intervention to an overly rigorous test, and future research might usefully include a larger and more diverse sample of children, from a wider range of schools. We also do not know how well or faithfully the interventions were implemented because the fidelity of implementation was not assessed in this initial study.

To the extent that the Mindfulness + Reflection group was better than BAU at Follow-up (the delayed post-test), there is support for the idea that combining mindfulness and reflection training may provide children with potentially valuable improvements in their EF skills. We were unable to parse the separate contributions of mindfulness, reflection, and practice with EF games in the present design, however, we hypothesize that reflection, which fosters an internal verbal commentary about one's actions vis-à-vis goals, is an essential ingredient that may be especially important for allowing transfer of trained EF skills to new situations and assessments (Espinet et al., 2013). Moreover, mindfulness may support reflection training by reducing emotional distress which can interfere with reflection and the top-down control of attention (Zelazo and Lyons, 2012). An important goal for future research will be to reveal the conditions under which interventions of this sort are maximally effective, and for whom. Future research should also address several limitations of the current study that make interpretation difficult. These include the lack of fidelity measures, the low parent participation rate in DC, and the lack of a longer-term follow-up assessment to examine possible positive cascades or fade-out effects.

## Conclusion

Interventions designed to reduce stress and increase reflection may have the potential to help children at risk for a wide range of difficulties. Research is growing on the efficacy of interventions designed to interrupt automatic responding and reflect on situations prior to acting, and there is evidence that the processes involved in reflection become more efficient with practice. Results of this study align with other evidence suggesting that it may be possible to target EF skills during the preschool years to improve school readiness. However, it is clear that further study is needed to elucidate optimal strategies for improving EF skills in high-risk preschoolers, as well as the key moderators of response to intervention. Effects were quite modest in this initial trial. Nonetheless, there were signs of positive change, particularly when measured 4 weeks following the end of the 6-week intervention. Further iterative research is needed to improve the curriculum employed here, consolidate and broaden the generalization of EF skills, study the fidelity of implementation and expand the indicators of response to intervention. Results also suggest that children should be followed for a longer period of time.

The preschool years may be a window of opportunity for the development of EF skills due to a combination of brain plasticity, rapid development of the neurocognitive processes supporting EF skills in this developmental window, and the growing prevalence of preschool attendance and scholarships for low-income children to gain access to high quality early childhood education (e.g., Zelazo, 2015). Basic scientific research on EF suggests that these skills have may have cascading effects on achievement and well-being (e.g., Carlson et al., 2013, for review). Intervention studies using randomized controlled trials offer the best strategy to test the feasibility and efficacy of initiating a positive cascade to success among very disadvantaged children (Masten and Cicchetti, 2010). This is an important and challenging research agenda that could yield high returns on investment.

## Author contributions

PZ, SC, AM, and JF designed the Mindfulness + Reflection intervention, and JF trained teachers to deliver it; PZ and SC designed the evaluation trial; SC and PZ were responsible for data analysis; PZ, SC, AM, and JF wrote the article.

### Conflict of interest statement

PZ, SC, and the University of Minnesota are entitled to royalties from the sale of the Minnesota Executive Function Scale (MEFS) by Reflection Sciences, Inc. The other authors declare that the research was conducted in the absence of any commercial or financial relationships that could be construed as a potential conflict of interest.
