# Supplementary material for: Mindfulness Plus Reflection Training: Effects on Executive Function in Early Childhood
Source: Front Psychol. 2018 Feb 26;9:208. doi: 10.3389/fpsyg.2018.00208 (PMC5834482; doi:10.3389/fpsyg.2018.00208)
Supplement: Supplementary file 1 [file DataSheet1.pdf]

Zelazo , P. D., Forston, J. L., Masten, A. S., & Carlson. S. M. (2018). Mindfulness plus reflection training: Effects on executive function in early childhood. *Frontiers in Psychology.*

Please address correspondence to: Philip David Zelazo, Institute of Child Development, University of Minnesota, 51 East River Road, Minneapolis, MN 55455-0345. E-mail: zelazo@umn.edu.

Appendix
Examples of exercises used in the Mindfulness + Reflection intervention

The 14 mindfulness exercises:

Lesson 1-- **Getting to Know You, Getting to Know Your Breath**
Lesson 2-- **The Breath in My Body**
Lesson 3-- **Introduction to Body Scan, Sound in Space**
Lesson 4-- **Body Scan and Mindfulness Journals**
Lesson 5-- **Mindful Movement and Deep Body Scan**
Lesson 6-- **Deep Body Scan and Mindfulness Journals, Cheerios box**
Lesson 7-- **Mindful Seeing**
Lesson 8-- **Mindful Hearing, Egg Carton Game**
Lesson 9-- **Mindful Touch, “What’s Behind My Back?” game**
Lesson 10-- **Mindful Smelling Mindfulness Journal blank pages**
Lesson 11-- **Mindful Taste**
Lesson 12-- **Interconnectedness book: *Who Put the Cookies in the Cookie Jar*?**
Lesson 13-- **Baking Soda in Water and *You Are Not Your Thoughts* book**
Lesson 14-- **Snow Globe/Glitter ball activity**

Each lesson begins with a greeting, and ends with friendly wishes and a song (Be Well). New practices are introduced that are relevant to the Lesson theme, and some previously introduced practices are repeated in later lessons. The first 3 lessons provide an example:

Lesson 1-- **Getting to Know You, Getting to Know Your Breath**
Greeting
Parachute breathing
Sleeping lions (Wemoweh song)
Be Well Song

Lesson 2-- **The Breath in My Body**

Greeting

Breathing ball

Pinwheel

Starfish

Sleeping beanies breath

Friendly wishes

Be Well Song

Lesson 3-- **Introduction to Body Scan, Sound in Space**

Greeting

Tic Toc

Breathing ball

Body scan with hula hoop

Sound in Space game (Tone bar)

Sleeping beanies breathe

Friendly wishes

Be Well Song

Sources:

Flook, L., Smalley, S. L., Kitil, M. J., Galla, B., Kaiser-Greenland, S., Locke, J., Ishijima, E., Kasari, C. (2010). Effects of mindful awareness practices on executive functions in elementary school children. *Journal of Applied School Psychology, 26*(1), 70-95.

Kaiser Greenland, S. (2010). *The mindful child: How to help your kid manage stress and become happier, kinder, and more compassionate*. New York: Free Press.

Saltzman, A. (2014). *A still quiet place: A mindfulness program for teaching children and adolescents to ease stress and difficult emotions.* Oakland, CA: New Harbinger.

Schoeberlein, D., & Koffler, T. (2005). *Garrison Institute report: Contemplation and education: A survey of programs using contemplative techniques in K–12 educational settings: A mapping report*. New York: Garrison Institute.

Willard, C. (2010). *Child's mind: Mindfulness practices to help our children be more focused, calm, and relaxed.* Berkeley, CA: Parallax Press.
